# Enhancing workforce diversity by supporting the transition of internationally educated nurses

**DOI:** 10.1097/01.NUMA.0000816252.78777.8f

**Published:** 2022-02-03

**Authors:** Kathy Rovito, Adam Kless, Shari Dingle Costantini

**Affiliations:** At Avant Healthcare Professionals in Casselberry, Fla., **Kathy Rovito** is the research director; **Adam Kless** is the vice president, clinical operations; and **Shari Dingle Costantini** is the founder.

## Abstract

Read about a study that explored nurse leaders' perspectives on the clinical performance of internationally educated nurses who completed a transitions program to support their acculturation to the US.

**Figure:**
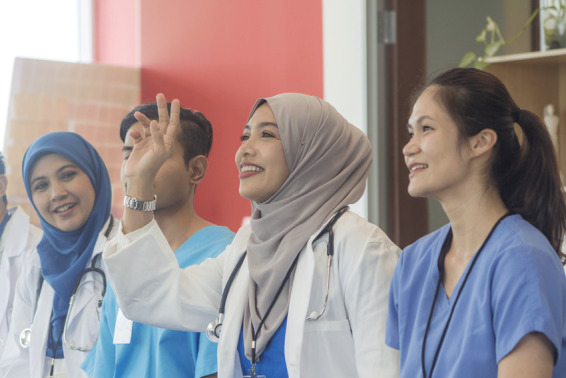
No caption available.

Internationally educated nurses (IENs) experience several cross-cultural barriers that can challenge their acculturation to the US and, in turn, the delivery of care. The purpose of this study was to assess the clinical performance of IENs who completed a transitions program to support their acculturation.

## Background

The premiere destination for international migration is the US, driven by the desire for better employment opportunities and working conditions.^1^ According to projections from the US Census Bureau, by the year 2030, immigration (versus natural increase) is expected to be the main driver of the country's population growth. Duly, the US is expected to become a majority-minority nation by 2045.^2^ In fact, the fastest growing racial or ethnic group in the US is people who identify as two or more races, followed by those who identify as Asian.^2^ Notably, by 2060, roughly one in four people living in the US will be foreign-born.^2^

### Diversity in the nursing workforce

Shifts in the nation's demographics present our healthcare system with challenges related to changes in the burden of disease, the delivery of care to patients with different healthcare beliefs and practices, and patient-provider concordance.^3^ Certain strategies are employed to assist with alleviating these issues, such as cultural competence and diversity sensitivity trainings within the workplace, among others.^3^ However, another strategy consists of enhancing the diversity of the nursing workforce through ethical and quality recruitment, staffing, and retention of IENs. There's emerging evidence to suggest that staffing IENs, also known as foreign-educated or foreign-trained nurses, can be beneficial to address these challenges and potentially improve the overall experience of the nursing workforce.^4^,^5^

According to a recent report by the International Council of Nurses, global migration of international healthcare professionals is increasing in both magnitude and complexity.^6^ Global data from 2018 to 2019 indicate that one out of every eight nurses practices in a country other than the one where they were born or trained.^7^ Since 2011, the number of IENs working across the 36 high-income Organisation for Economic Co-operation and Development countries increased by 90,000.^6^ The US is the leading destination country, home to approximately 36% of the globally registered IENs.^6^ In 2018, of the 3.96 million RNs in the US, approximately 5% completed their primary nursing education training outside of the US.^8^

The need for diversity in nursing has been well articulated by many leading national organizations to address culturally competent, patient-centered care and reduce health inequities.^9^,^10^ Diversifying the healthcare workforce has the potential to improve healthcare delivery from a business and patient outcome standpoint. Although there's a paucity of robust research linking diversity in healthcare and clinical outcomes, there's a growing body of evidence showcasing the corporate benefits of a diverse workforce.^11^ For example, greater diversity in the workforce is positively linked to several financial performance measures, including productivity, risk assessment precision, and innovation.^12^-^16^ Limited research also suggests that greater healthcare workforce diversity can be used as a vehicle to improve patient-provider interactions and, in turn, improve patient outcomes and reduce health disparities.^17^-^19^

### Do IENs have the skills to work in this environment?

Growing literature demonstrates that IENs are an important part of the US nursing workforce.^20^ Further, employing IENs may help mitigate the ongoing nursing shortage exacerbated by the COVID-19 pandemic.^21^ However, concerns are common among potential employers related to the clinical preparation of IENs. One prevailing concern stems from the fact that IENs complete their primary nursing education and training outside of their current country of employment.^5^ The standards for safety skills and overall quality, as well as expectations for practice, may be construed differently across cultures.

Considering the above concern, a major component of the regulation governing IEN migration is ensuring baseline safety and quality standards for practice, including educational comparability and nursing licensure.^5^ Due to the complexity of the credentialing process, which involves navigating governing and legal systems, IENs experience struggles and setbacks with gaining entry into and practicing in the US.^5^

International experiences also come with inherent benefits to nursing practice that may distinguish IENs from novice or new graduate nurses without such experience. The literature demonstrates that IENs mobilize their linguistic and international nursing practices as assets to transcend the challenges and new experiences in their new home country and work environment.^22^ Other research indicates international experiences in nursing bring about a higher level of awareness and sensitivity to people's differences that may influence how nurses recognize and respond to different cues for underlying concerns.^23^ In other words, the IEN's life and work experiences may provide them with a particular set of skills that contribute to a maturity with interpersonal communication and nursing knowledge.

Evidence highlighting IENs' influence on clinical outcomes is limited. Some research indicates a negative association between hospitals employing more IENs and the patient care experience.^24^ However, other research suggests that employing more IENs doesn't negatively affect collaboration among healthcare professionals and, in fact, results in a more stable and educated nursing workforce.^25^ More studies are needed in this area to further examine the influence that IENs have on patient care in the US.

### Facilitating acculturation of IENs to the US healthcare system and mainstream society

Culture is a fundamental building block of place and personal identities.^26^ It plays a critical role in who we are, how we view the world, and how we interact with it and with one another. As IENs migrate to the US, they bring with them their own cultural identity, which contributes to the diversity of our society and the nursing workforce. Acculturation describes how IENs adapt culturally as they interact with the new culture. The acculturation process of an IEN involves adopting normative practices, behaviors, attitudes, and values of the US mainstream and nursing culture, while allowing the space to maintain their own identity tied to their home culture.^27^

Cultural exchange is a proverbial “two-way street” where each group or society gives to and takes from the other, thus strengthening both. However, it isn't without growing pains. Previous studies demonstrate that cultural differences between an IEN and their host country introduce both personal and professional challenges, thus affecting the transition experience and acculturation. The most common challenges experienced during transition are barriers related to language and communication, such as colloquialisms and body language interpretation, and racism and discrimination, such as marginalization due to being seen as an outsider.^28^ Additionally, differences in nursing practice, such as clinical decision-making, electronic medical record usage, and assertiveness and delegation, are the most stressful barriers for IENs during their transition to the US work environment.^4^

The risks associated with such cultural dissonance are detrimental to both nurses and patients. For example, these barriers may trigger social isolation and emotional and mental distress, which can exacerbate marginalization for the nurse and further lead to issues with the delivery of care and patient safety.^4^,^28^ Plainly, IENs are expected to navigate the complex cross-cultural challenges in a foreign country. Their ability to fill the role of a nurse to the standards and expectations of the US is dependent on how well they cope with these challenges. Therefore, how IENs are supported through this transitionary time is of critical importance.

Culturally sensitive support measures for IENs are essential to the advancement of nursing, the employer, patients, and nurses' health and well-being. Empirical evidence suggests that IENs who successfully acculturate to their host culture report greater satisfaction with life and their profession.^29^ There's also evidence suggesting that RNs, domestically or internationally educated, stay in their profession longer and contribute to better patient outcomes when they're satisfied with their personal lives and careers.^30^

Considering the aforementioned concerns, this study sought to explore nurse leaders' perspectives on the clinical performance of IENs who have completed a transitions program, which aims to support the acculturation of IENs to the US. Outcome measures included cross-cultural clinical interaction performance ratings.

## Methods

As part of the effort to support IENs' migration to the US, a Joint Commission-accredited recruitment and staffing agency offers a personalized and comprehensive, 5-week clinical and cultural transitions program. This transitions program is offered to each IEN candidate before their job assignment as they transition to their new healthcare team and new home in the US. IENs are primarily placed in acute care facilities across the US and skill- and experience-matched to specific facility needs.

The program includes training on the clinical and cultural differences of practicing in the US compared with the IEN's home country. Training is provided through in-person live lectures, lab activities, role playing, and clinical simulation equipment. The American Association of Critical-Care Nurses Synergy Model for Patient Care is the foundation for the clinical portion of the transitions program. Topics include electronic health records and documentation, team communication, pharmacology, Hospital Consumer Assessment of Healthcare Providers and Systems surveys, and Medicare and third-party payers. IENs also receive hands-on practice with clinical equipment, such as smart I.V. pumps, surgical instruments, lines, drains, and medication dispensing cabinets. Additional content is covered if the IEN is being assigned to a specialty area.

The program also includes a curriculum on the stages of culture shock to help the IEN identify and appropriately navigate the phases of adjustment to their new home and work environment. Additional logistical support, such as assistance in acquiring a social security number, housing, transportation, or schooling for children, is also offered to IENs. Once the transitions program is completed and the IEN is placed on assignment, they receive ongoing coaching and mentorship throughout their 1.5- to 2-year assignment.

Most studies on IENs are through the lens of the IEN; however, it's critical to understand the nurse leader perspective. Nurse leaders who are responsible for hiring and/or supervising IENs are the gatekeepers in charge of granting IENs permanent employment in the US and ensuring a work environment that allows the nurse to thrive as a US nurse.^5^ Accordingly, this exploratory, cross-sectional study sought to understand nurse leaders' perspectives regarding the clinical performance of a sample of IENs who completed the recruitment and staffing agency's transitions program.

To accomplish this, a survey designed and implemented in 2019 evaluated the performance of IENs who completed the transitions program and the first 90 days of their job assignment. Nurse leaders with direct knowledge of the performance of IENs were recruited from client facilities to participate. Inclusion criteria consisted of: self-identified as a nurse manager or other nurse leader (such as nurse educator), supervised at least one IEN who completed the transitions program, and completed proper informed consent to participate. There were no additional exclusion criteria. Procedures for the study were approved by the Institutional Review Board.

The three overarching cross-cultural clinical interaction measures assessed in the study were communication, caring practices, and core nursing care. The communication section of the survey included four items about how IENs facilitate a patient's understanding of their delivery of care. The caring practices section included three items related to patient advocacy and empowerment. The core nursing care section included seven items about care delivery skills associated with nursing; for example, answering call lights. The items were scored on a 7-point Likert scale assessing level of agreement from 1 (absolutely don't agree) to 7 (absolutely agree), with higher scores indicating a more positive rating of performance.

Study measures were identified by a panel of experts from the field of nursing and international healthcare staffing as areas that emphasize a congruent set of factors to enable IENs to practice effectively in cross-cultural clinical situations in the US. This expert assessment ensured content validity of the measures. The items underlying each of the overarching measures demonstrated acceptable internal consistency as represented with Cronbach's alpha scores of .63, .79, and .69, respectively.

Nurse leader participants were also asked to self-report social-demographic and occupational characteristics, including their age, gender, race/ethnicity, highest level of education, length of time in current leadership position, and previous experience working with or as an IEN. In addition to the IEN performance ratings, participants were also asked to report the home country of the IEN they were evaluating.

## Results

There were 64 nurse leaders from clinical facilities throughout the US who took part in the study. In this study, most participants were ages 41 to 50 (39.1%, n = 25), female (92.2%, n = 59), and identified as White/non-Hispanic (87.5%, n = 56). The highest level of education for most participants was a bachelor's degree in nursing (42.2%, n = 27). Many participants were in their nurse leader position for 1 to 4 years (50%, n = 32), supervised at most two IENs per day (57.8%, n = 37), and had no previous experience working with or as an IEN (73.4%, n = 47). Most IENs evaluated were from Jamaica (45.3%, n = 29), followed by the Philippines (21.9%, n = 14).

Table 1 shows the mean performance ratings for the IENs who completed the transitions program. The overall results indicate highly favorable evaluation responses across communication, caring practices, and core nursing care, with the most favorable ratings given to questions about paraphrasing, hourly rounding, and bedside shift reports. The data also demonstrate that there's an opportunity to support IENs in US nursing decision-making skills.

**Table 1:** Nurse leader ratings on the clinical performance of IENs (N = 64)

Item	Mean∗	SD
**Communication**		
Demonstrates satisfactory decision-making skills.	5.02	0.85
Uses therapeutic communication with patients.	6.01	0.74
Uses key words to help patients better understand their care plan and treatment regimen.	5.94	0.79
Paraphrases to seek patient understanding.	6.14	0.79
**Caring practices**		
Honors and reinforces the patient's autonomy by asking permission to provide care before services.	6.08	0.65
Individualizes care for each patient and their family.	5.93	0.77
Validates patient cultural sensitivities.	5.80	0.89
**Core nursing care**		
Readdresses the “three p's” (pain, potty, and position) for each patient during hourly rounding.	6.12	0.71
Participates in effective bedside shift report.	6.14	0.94
Manages patient's pain expectations.	6.03	0.91
Addresses pain during bedside shift report.	5.89	0.88
Reinforces medication education during bedside shift report.	5.42	1.26
Demonstrates bathroom assistance as a critical aspect of their role.†	5.99	0.91
Demonstrates answering call lights as a critical aspect of their role.†	5.91	0.96

Note: SD = standard deviation

∗ Item responses based on a 7-point Likert-type scale for level of agreement, with higher scores indicating a more positive rating of performance.

† IENs are primarily placed in acute care facilities. Therefore, responsiveness to toileting and call lights is important.

## Discussion

Preliminary findings from this exploratory study indicate that nurse leaders think highly of IENs who complete the transitions program. The results suggest that nurse leaders believe IENs who complete the transitions program perform well in areas of communication, caring practices related to patient advocacy and empowerment, and core nursing care (bedside skills). Although the mean ratings are positive, demonstrating satisfactory decision-making skills received the lowest average rating of all the performance areas assessed. This finding is in line with previous research indicating variations in nursing autonomy as a significant cross-cultural challenge for IENs.^4^ When supporting the transition of IENs to the US, it's important to recognize that improvements with many of these cross-cultural challenges don't happen overnight. Therefore, sustainable support methods are needed to facilitate IEN acculturation to the US.

Supporting the transition of IENs to the US is a multidimensional, intersectional, and bidirectional process that should place healthcare discourse within a cultural context. A transitions program is designed to bridge the gaps in practice from the IEN's home country and the US. Although the IEN receives a full orientation to their new facility and unit based on the specialty area, this orientation is meant to be no different from what any new US-educated nurse would get at that facility. The length of orientation will vary based on the specialty area, the level of acuity, and the nurse's individual needs. The IEN typically needs a different orientation than a US-educated nurse. An important consideration when providing orientation to an IEN is their possible need to unlearn aspects of nursing practice that were common in their home country but aren't part of US nursing practice.

## Implications for nursing management

As globalization continues to be a key determinant of population dynamics worldwide, more IENs will be working in US healthcare facilities. Concurrently, the COVID-19 pandemic has exacerbated the ongoing nursing shortage and workforce demands, directly hindering the capacity to deliver quality healthcare. The pandemic has pushed many nurses to the verge of burnout, with approximately one in three nurses considering leaving the bedside.^31^ This is critical because nurse-to-patient ratios are directly linked to patient safety.^31^ Staffing IENS can help mitigate the challenges many healthcare facilities are experiencing with a fatigued workforce.

Nurse leaders and healthcare organizations have a responsibility to address the barriers preventing these highly skilled nurses from making a smooth transition to their new home and workplace. Study findings indicate we should celebrate the benefits of diversity and the role that IENs have in not only enhancing the diversity of the nursing workforce but also stabilizing it. Part of these efforts include nurturing and leveraging the IEN's experience so they can apply it to the US nursing paradigm.

A transitions program is instrumental in providing the needed support for IENs by bridging the gap between clinical and cultural transitions training while offering personalized, ongoing support to promote cultural congruence in our healthcare system. The newly released report *The Future of Nursing 2020-2030* echoes these calls, specifically in its fourth and eighth recommendations related to dismantling barriers that prevent nurses from practicing to their full potential and strengthening the nursing workforce to better respond to public health crises, respectively.^9^

## Limitations

As with most studies, this study has limitations. First, there may be other factors external to the transitions program, such as characteristics of the IEN or variables unique to the assessment period, that weren't considered in this study but could affect the IEN's clinical performance. Second, the subjective evaluation of the IEN's clinical performance should be interpreted with caution. Future studies should triangulate the data with more objective measures, such as direct observations, chart reviews, or 360-degree peer feedback. Third, due to the exploratory nature of this descriptive study, the study measures didn't undergo comprehensive psychometric testing. Future studies should conduct rigorous validity and reliability assessments.

## Recognizing the value of IENs

This exploratory study provides a framework for the transitional support of IENs. The findings demonstrate that with the appropriate transitional support, which can be achieved by partnering with organizations that support IENs in the workplace, nurse managers shouldn't be reluctant to staff IENs in their healthcare facility. Nurse leaders should recognize the contributions of their IEN colleagues and the value they add to the immediate work environment and the nursing workforce.

## Research overview

**Purpose:** To explore nurse leaders' perspectives on the clinical performance of IENs who completed a transitions program, which aims to support the acculturation of IENs to the US

**Location:** Online survey disseminated to clients of a recruitment and staffing agency throughout the US

**Time frame:** July to October 2019

**Population:** Nurse leaders with direct knowledge of the performance of IENs who completed the transitions program

**Data collected:** Cross-cultural clinical interaction measures: communication, caring practices, and core nursing care

**Sample size:** N = 64
